# Epigallocatechin-3-Gallate, the Main Polyphenol in Green Tea, Inhibits Porcine Epidemic Diarrhea Virus *In Vitro*

**DOI:** 10.3389/fphar.2021.628526

**Published:** 2021-02-22

**Authors:** Changchao Huan, Weiyin Xu, Bo Ni, Tingting Guo, Haochun Pan, Luyao Jiang, Lin Li, Jingting Yao, Song Gao

**Affiliations:** ^1^ Institutes of Agricultural Science and Technology Development, College of Veterinary Medicine, Yangzhou University, Yangzhou, China; ^2^ Jiangsu Co-Innovation Center for Prevention and Control of Important Animal Infectious Diseases and Zoonoses, Yangzhou, China; ^3^ Key Laboratory of Avian Bioproduct Development, Ministry of Agriculture and Rural Affairs, Yangzhou, China; ^4^ China Animal Health And Epidemiology Center, Qingdao, China; ^5^ College of Medicine, Yangzhou University, Yangzhou, China

**Keywords:** porcine epidemic diarrhea virus, epigallocatechin-3-gallate, green tea polyphenol, drug, virus inhibition

## Abstract

There are currently no licensed drugs against porcine epidemic diarrhea virus (PEDV), but vaccines are available. We identified a natural molecule, epigallocatechin-3-gallate (EGCG), the main polyphenol in green tea, which is effective against infection with PEDV. We used a variety of methods to test its effects on PEDV in Vero cells. Our experiments show that EGCG can effectively inhibit PEDV infections (with HLJBY and CV777 strains) at different time points in the infection using western blot analysis. We found that EGCG inhibited PEDV infection in a dose-dependent manner 24 h after the infection commenced using western blotting, plaque formation assays, immunofluorescence assays (IFAs), and quantitative reverse-transcriptase PCR (qRT-PCR). We discovered that EGCG treatment of Vero cells decreased PEDV attachment and entry into them by the same method analysis. Western blotting also showed that PEDV replication was inhibited by EGCG treatment. Whereas EGCG treatment was found to inhibit PEDV assembly, it had no effect on PEDV release. In summary, EGCG acts against PEDV infection by inhibiting PEDV attachment, entry, replication, and assembly.

## Introduction

Porcine epidemic diarrhea (PED) is characterized by acute villus atrophy and congestion, severe watery diarrhea, dehydration and death. PED can infect pigs of all ages and causes high mortality in newborn piglets (mortality rate is nearly 100%) (Li, et al., 2012; Zhang, et al., 2019). Its causative agent is porcine epidemic diarrhea virus (PEDV). PEDV was first recognized in 1971 in England (Wood, 1977; Pensaert and de Bouck, 1978). Although PED has been reported in Asia since the 1980s, its prevalence has been comparatively low (Takahashi, et al., 1983; Puranaveja, et al., 2009; Lin, et al., 2014; Jung, et al., 2016; Sun, et al., 2016). Since late 2010, the new PED strains with high pathogenicity in China have been regarded as pandemic strains (Sun, et al., 2016). In 2013, highly pathogenic PEDV was first seen in the USA where it quickly spread to the neighboring countries such as Canada and Mexico (Mole, 2013; Stevenson, et al., 2013; Chen, et al., 2014; Lowe, et al., 2014; Vlasova, et al., 2014). To date, PED reoccurrence has become more common in pigs immunized with a commercial vaccine (Li, et al., 2012; Sun, et al., 2012; Tian, et al., 2013). Therefore, PEDV continues to spread widely and cause huge economic losses to the swine industry. With the prevention and control of PED now very urgent, we have focused our attention on the potential use of traditional Chinese medicine against PEDV.

PEDV is a member of alpha coronavirus in the Coronaviridae family, and it possesses a single-stranded positive-sense RNA genome about 28 kb (Kocherhans, et al., 2001). PEDV size ranges in diameter from 95 nm to 190 nm. PEDV genome includes a 5’ untranslated region (UTR)，two overlapping open reading frames (ORFs) encoding two polyproteins (ORF1a and ORF1b), ORFs 2–6 encoding four major structural proteins (spike (S), ORF3, envelope (E), membrane (M), and nucleocapsid (N)) and one accessory ORF3 protein (Duarte, et al., 1993; Lee and Lee, 2010).

Green tea is consumed as a drink in worldwide (Hayat, et al., 2015). In 2017, the production of global tea was 5.686 million tons, while the production of China’s tea was 2.609 million tons. Epigallocatechin-3-gallate (EGCG) is a natural compound in green tea, and EGCG accounts for about 59% of the total catechin in it. Other catechins in green tea include epicatechin (EC) (6.4%), epicatechin gallate (ECG) (13.6%), and epigallocatechin (EGC) (19%) (McKay and Blumberg, 2002). Structurally and functionally, the properties of catechin are attributed to the presence or absence of a galloyl moiety and the number of hydroxyl groups on its B-ring. EGCG displays potent inhibitory effects toward human immunodeficiency virus, influenza virus, hepatitis virus, hepatitis B virus, porcine reproductive and respiratory syndrome virus and porcine circovirus type 2 (Fassina, et al., 2002; Yamaguchi, et al., 2002; Jariwalla, et al., 2007; Roomi, et al., 2008; Fukazawa, et al., 2012; Calland, et al., 2012; Chen, et al., 2012; Ge, et al., 2018; Li, et al., 2020). However, whether EGCG has an inhibitory effect on PEDV has not been reported. Therefore, we evaluated whether EGCG inhibited PEDV infection.

## Results

### EGCG Inhibits PEDV Infection

**FIGURE 1:**
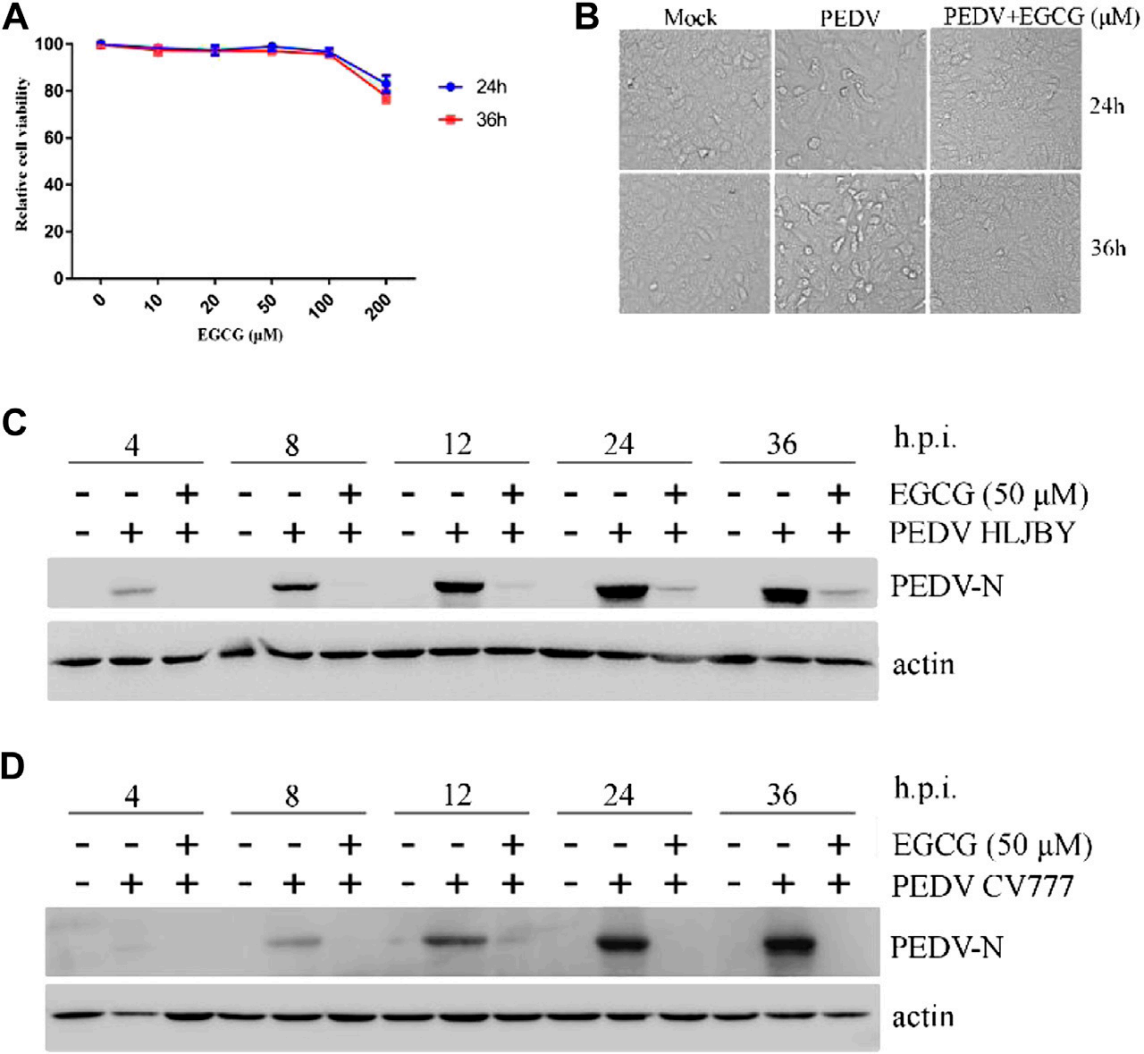
EGCG inhibition of PEDV at different time points. **(A)** Cytotoxicity assay using soluble EGCG. EGCG concentrations (0, 10, 20, 50, 100 and 200 μm) were added to Vero cells and the cells were cultured for 24 h–36 h. Cell viability was evaluated by the CCK8 assay and calculated as (A450 compound/A450 mock) × 100%. **(B–D)** EGCG inhibits PEDV infection. Vero cells were pretreated with different concentrations of EGCG for 1 h and then infected with PEDV HLJBY (0.1 MOI) or PEDV CV777 (0.1 MOI) for 4, 8, 12, 24 and 36 h in the presence of EGCG. **(B)** EGCG treatment decreased the cytopathic effect on the cells at 24 and 36 h. **(C,D)** PEDV N protein expression levels were detected by western blotting at different time points.

**FIGURE 2:**
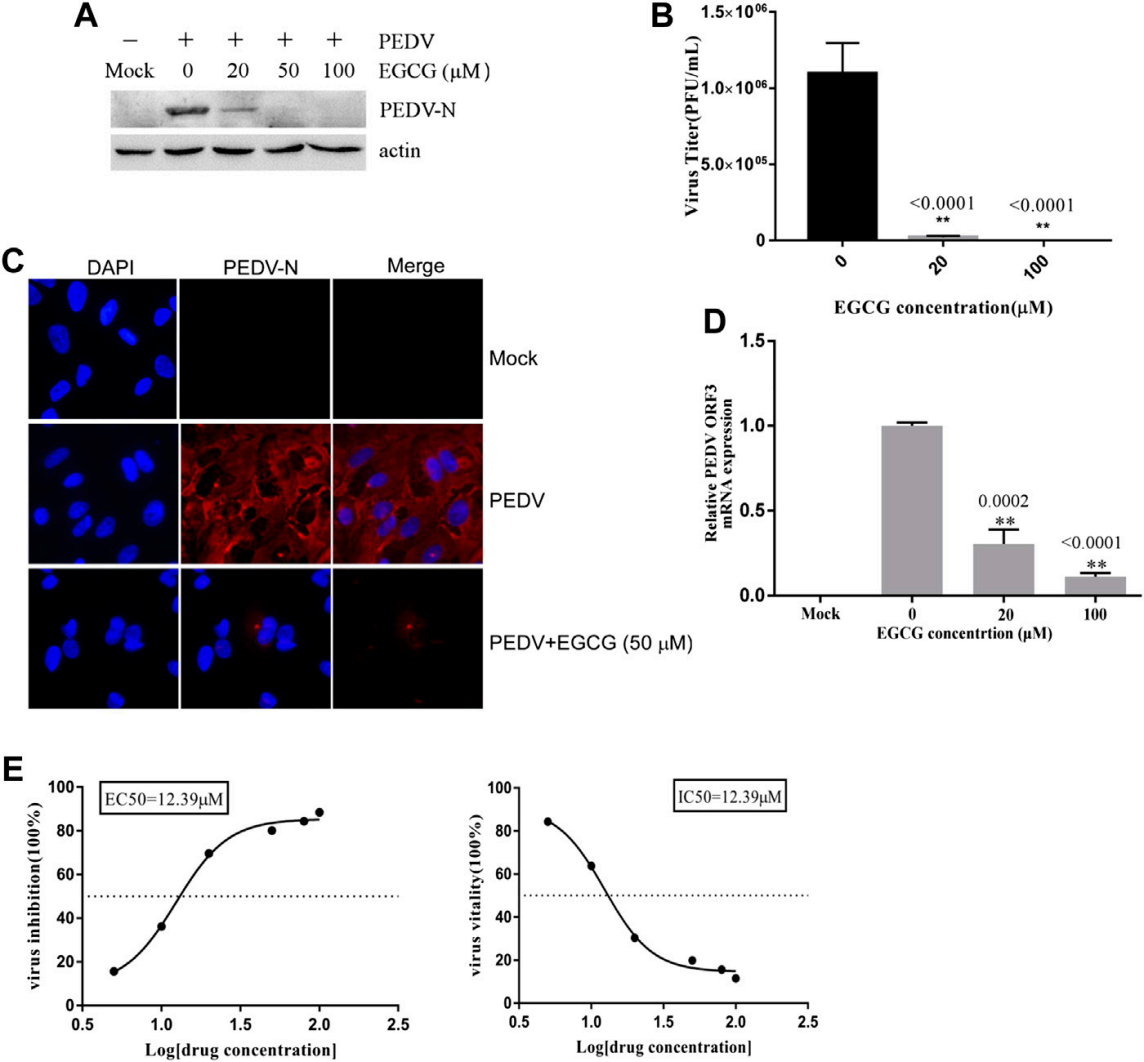
EGCG exhibits an anti-viral effect on PEDV infection in Vero cells. Cells were pre-treated with different concentrations of EGCG for 1 h before being infected with PEDV HLJBY (0.1 MOI) and were then treated with different concentrations of EGCG for 24 h. Intact cells and supernatants were collected at 24 h.p.i. **(A)** PEDV N and actin proteins were detected by western blotting. **(B)** Viral titers were determined using a plaque formation assay. **(C)** IFA detection of PEDV infected cells. **(D)** PEDV mRNA was quantified by qRT-PCR. **(E)** EC50 or IC50 of EGCG against PEDV infection was checked.

To assess the antiviral activity of EGCG against PEDV, the cytotoxicity of EGCG in Vero cells was first evaluated using a CCK8 assay. The results revealed that when used for 24 and 36 h at 100 μM, EGCG was not cytotoxic to Vero cells, whereas it was cytotoxic at 200 μm (Figure 1A). Therefore, the maximum concentration of EGCG used in the experiments was 100 μm. Vero cells were pretreated with different concentrations of EGCG for 1 h and then infected with PEDV HLJBY (0.1 MOI) or PEDV CV777 (0.1 MOI) for different time periods with EGCG present. EGCG treatment decreased the cytopathic effect of PEDV HLJBY on the cells (Figure 1B). In addition, western blotting showed that PEDV HLJBY and PEDV CV777 N protein expression levels decreased significantly at the different time points (Figures 1C, D). Therefore, we determined the inhibitory effect of PEDV HLJBY infection on the cells for the 24 h period by western blotting, plaque formation assays, IFAs and qRT-PCR. Western blotting showed that the expression level of the PEDV HLJBY N protein decreased significantly under different concentrations of EGCG, and the decrease in the protein level was approximately 99.6 and 99.9% at 50 μm and 100 μm of EGCG, respectively (Figure 2A). The inhibition of PEDV HLJBY infection was approximately 99.9% at 100 μm of EGCG, as demonstrated by the decreased viral titers detected by the plaque formation assay (Figure 2B). The number of cells infected with PEDV HLJBY was clearly lower than that of the controls in the IFA (Figure 2C), and the reduction of the PEDV HLJBY ORF3 was about 93.2% at 100 μm EGCG by qRT-PCR (Figure 2D). In addition, the EC50 or IC50 of the antiviral effect of EGCG on PEDV HLJBY was further determined to be 12.39 µm (Figure 2E). These results confirm that EGCG displays marked antiviral activity against PEDV infections.

### EGCG Impairs PEDV Attachment to Vero Cells

**FIGURE 3:**
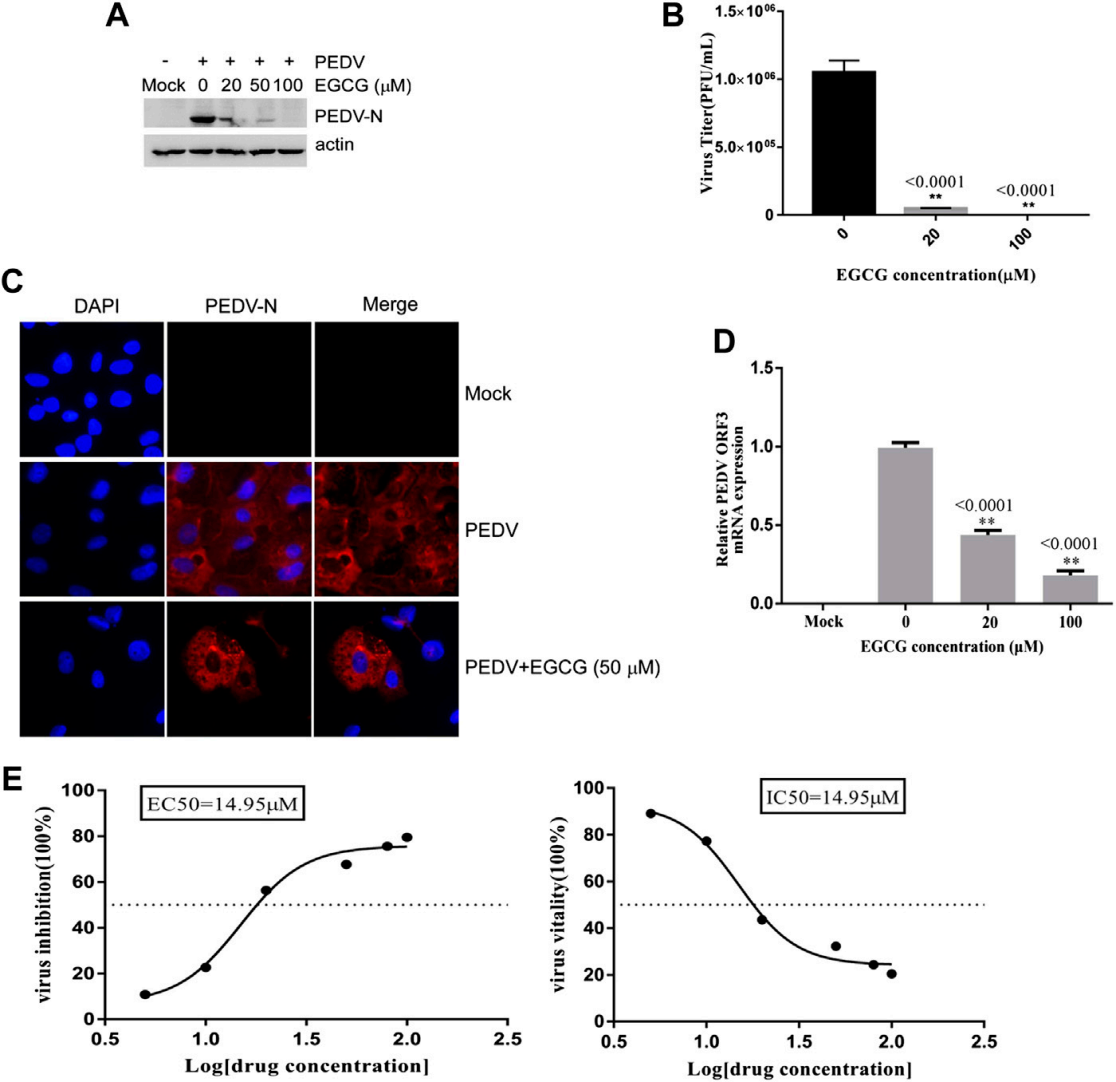
EGCG prevents PEDV binding to Vero cells. **(A–C)** Vero cells were pretreated with EGCG for 1 h and then infected with PEDV HLJBY at 4°C for 1 h with EGCG present. The cells were washed three times with PBS and then cultured at 37°C for 23 h. **(A)** PEDV N protein levels were detected by western blotting. **(B)** Supernatant-specific PEDV titers were assayed by a plaque formation assay. **(C)** The number of cells infected with PEDV was evaluated by IFA. **(D)** Vero cells were pre-treated with different concentrations of EGCG for 1 h before being infected with PEDV (0.1MOI) at 4°C for 1 h with EGCG present. The cells were collected to assay the level of PEDV ORF3 mRNA by qRT-PCR at 1 h.p.i. **(E)** EC50 or IC50 of EGCG against PEDV binding was checked.

To investigate the stage at which EGCG exerts its antiviral effect in the PEDV infection process, we first explored the effect of EGCG treatment on PEDV attachment to Vero cells. A binding assay was performed that involved pretreating Vero cells with EGCG for 1 h before infecting them with PEDV HLJBY at 4°C for 1 h with EGCG present. The cells were then washed with PBS 3 times and cultured at 37°C for 23 h. The western blotting results showed that EGCG inhibited the expression level of PEDV N protein, with an inhibition rate of 68.5–99.4% in an EGCG dose-dependent manner (Figure 3A). EGCG treatment also decreased the numbers of cells infected with PEDV, as determined by IFA (Figure 3C). Supernatants were collected for assaying the viral titers, which revealed that the PEDV titer fell by approximately 99.3% at 100 μM EGCG, as determined by the plaque formation assays (Figure 3B). Cells were also collected to determine the level of PEDV ORF3 mRNA by qRT-PCR, which showed that EGCG treatment significantly impaired the level of PEDV ORF3 mRNA (by about 53 and 79.2% at 20 and 100 μm, respectively) (Figure 3D). In addition, the EC50 or IC50 of EGCG inhibited attachment was 14.95 µm (Figure 3E). These results show that EGCG treatment reduced the attachment of PEDV to the Vero cells such that PEDV was unable to infect them.

### EGCG Affects PEDV Entry Into Vero Cells

**FIGURE 4:**
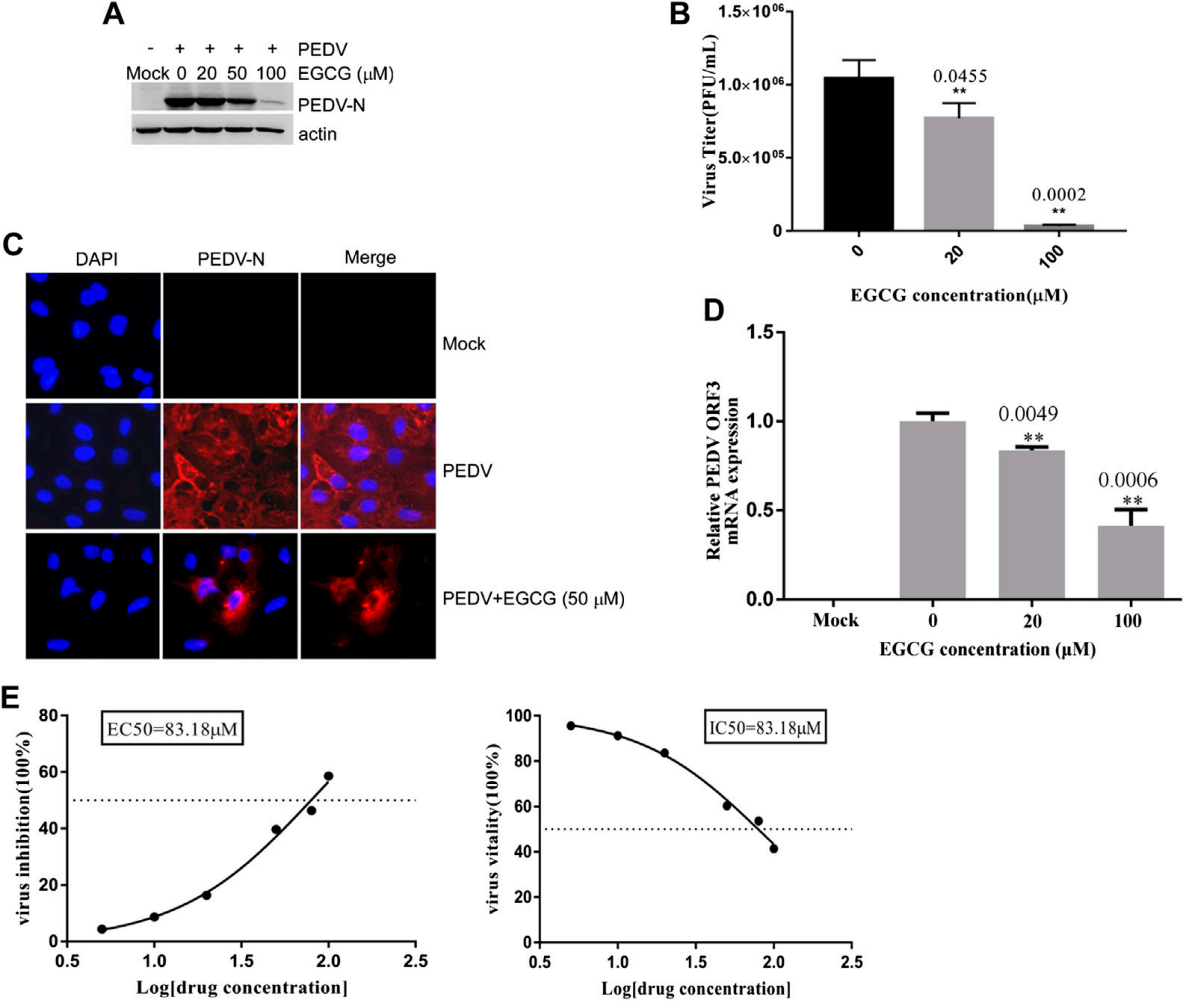
EGCG inhibition of PEDV entry into Vero cells. **(A–C)** Vero cells were infected with PEDV HLJBY with EGCG present at 37°C for 1 h. After 1 h the cells were washed three times with citric acid and three times with PBS, and 2% DMEM was added. The culture was allowed to continue for 23 h at 37°C. **(A)** The level of PEDV N protein was evaluated by western blotting. **(B)** Plaque formation assay for PEDV titers in the supernatants. **(C)** The number of cells infected with PEDV was evaluated by IFA. **(D)** When the Vero cells were infected with PEDV with EGCG present at 37 C for 1 h, the PEDV ORF3 mRNA levels were detected by qRT-PCR. **(E)** EC50 or IC50 of EGCG against PEDV entry was checked.

To explore whether EGCG is able to inhibit PEDV entry, Vero cells were infected with PEDV HLJBY in the presence of EGCG at 37°C for 1 h. After 1 h, the cells were washed three times in citric acid and three times in PBS, and 2% DMEM was added. The culture was allowed to continue for 23 h at 37°C. We found that EGCG reduced the level of PEDV N protein (by about 10–89.4%), as judged by western blotting (Figure 4A). The cells infected with PEDV were examined by IFA (Figure 4C). Supernatants were also collected to assay the viral titers, and the plaque formation assays revealed that the PEDV titers fell significantly by about 96.5% at 100 μm (Figure 4B). When the Vero cells were infected with PEDV with EGCG present at 37°C for 1 h, the PEDV ORF3 mRNA levels fell by about 81.1 and 56.7% at 20 μm and 100 μm, respectively (Figure 4D). In addition, the EC50 or IC50 of EGCG inhibited entry was 83.18 µm (Figure 4E). These results confirm that EGCG can block PEDV entry into Vero cells.

### The Effect of EGCG on PEDV Replication, Assembly and Release in Vero Cells

**FIGURE 5:**
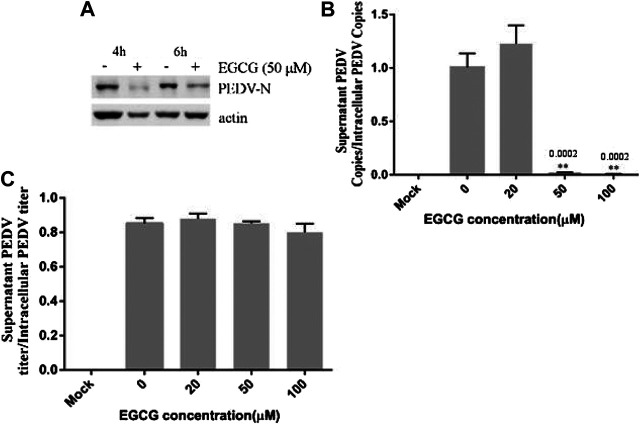
EGCG treatment decreased the replication and assembly of PEDV. **(A)** Vero cells were infected with PEDV HLJBY for 1 h and then treated with EGCG for 3 or 5 h. Western blotting analysis of PEDV N levels. **(B,C)** Vero cells were infected with PEDV HLJBY (0.1 MOI) at 37°C for 1 h before EGCG treatment. **(B)** The supernatants from the cells and the intact cells were collected to determine the copy number of the PEDV ORF3 gene at 24 h.p.i., as well as the PEDV titer ratio in the supernatant and in intact cells. **(C)** The collected supernatants and intact cells were used to determine the PEDV titers, from which the PEDV titer ratios in the supernatants to those of the intact cells were calculated.

To clarify the effects of EGCG on PEDV infection, Vero cells were infected with PEDV HLJBY for 1 h and then treated with EGCG for 3 and 5 h. Cells were collected to evaluate the expression levels of the PEDV N protein at 4 and 6 h.p.i. Western blotting showed that EGCG treatment decreased the level of the PEDV N protein (Figure 5A), suggesting that EGCG can prevent PEDV replication.

To investigate whether EGCG treatment affected viral assembly, Vero cells were infected with PEDV HLJBY (0.1 MOI) at 37°C for 1 h before adding EGCG. The cell supernatants and intact cells were collected to determine the copy numbers of the PEDV ORF3 mRNA at 24 h.p.i. The copy number ratio of the mRNA from the PEDV ORF3 gene in the supernatant to that of the PEDV ORF3 mRNA in the cells showed that EGCG inhibited PEDV assembly by about 99.6% at 100 μm (Figure 5B). In contrast, we found that the collected supernatant and intact cells that we used to determine the PEDV titer showed the opposite effect, whereby the ratio of PEDV titer in the supernatant to that of the PEDV titer in the cells showed that EGCG treatment had no effect on PEDV release, as evidenced by the results (Figure 5C).

## Materials and Methods

### Cells and Virus

Vero cells were cultured in Dulbecco’s modified Eagle’s medium (*Sigma-Aldrich*) supplemented with 8% fetal bovine serum (*Lonsa*) at 37°C with 5% CO_2_. PEDV HLJBY isolated from the intestinal contents of a pig-let with diarrhea from Heilongjiang province, and PEDV reference strain CV777 was purchased from China Institute of Veterinary Drug Control. PEDV HLJBY and PEDV CV777 caused pigs diarrhea, and acute viral enteritis with villous atrophy (Huan, et al., 2020). PEDV HLJBY and PEDV CV777 were stored at −80°C in the Yangzhou University Infectious Diseases laboratory.

### Reagents

EGCG was purchased from Selleck (China) and was diluted to stock solutions of 50 mm with PBS and stored at −80°C for all subsequent experiments. The purity of EGCG was 99.68% by HPLC assessed. The PEDV N antibody used herein was generated in our laboratory. The anti-PEDV-N polyclonal antibody was prepared in rabbits using PEDV-N protein as antigen and western blot and IFA were diluted 1:5,000 and 1:500, respectively (Gao, et al., 2020). The β-actin antibody was purchased from TransGenBiotech (China). HRP-labeled Goat Anti-Mouse IgG (H + L) and DAPI (4′,6-diamidino-2-phenylindole) was purchased from Beyotime Biotechnology (China). The citric acid solution (PH 3.0) is composed of 40 mm citric acid, 10 mm KCl, and135 mm NaCl, to remove un-internalized virus particles.

### Cytotoxicity of EGCG Toward Vero Cells

The cytotoxicity of EGCG toward Vero cells was evaluated following the manufacturer’s instructions from the CCK8 kit (Beyotime Biotechnology). Vero cells were seeded in a 96-well plate, and then treated with 10, 20, 50, 100, 200 μm concentrations of EGCG for 24–36 h. After 24–36 h of culture, 10 μl of CCK-8 solution per well was added and the plate was further incubated for 2 h at 37°C. Then we checked the absorbance at 450 nm to determine the maximum concentration of EGCG that was not toxic to the Vero cells.

### Cell-Based Assays

#### Infectivity Assay

We checked the life cycle of PEDV (MOI = 1) in Vero cells. We collected supernatants to check viral titer at hourly intervals between 1 and 12 h. Vero cells were pre-treated with EGCG (0, 20, 50,100 μm) for 1 h and then infected with PEDV (0.1 multiplicity of infection; MOI) for 4, 8, 12, 24, and 36 h with EGCG present. Vero cells were also pre-treated with different concentrations of EGCG for 1 h, followed by infection with PEDV (0.1 MOI) for 24 h with EGCG. Cells were collected to determine whether any changes had occurred in the N protein or the ORF3 mRNA of PEDV by western blot analysis and quantitative reverse–transcriptase quantitative PCR (qRT-PCR), respectively. Cell supernatants were collected to measure viral titers using a plaque formation assay.

#### Attachment/Binding Assay

Vero cells were pre-treated with different concentrations of EGCG (0, 20, 50,100 μm) for 1 h, followed by washing with cold PBS three times. The cells were then infected with PEDV HLJBY (0.1 MOI) at 4°C with the corresponding concentrations of EGCG for 1 h. The cells were washed in cold PBS three times (Norkin, 2009; Huan, et al., 2015). The cells and cell supernatants were then collected to evaluate PEDV N protein levels and determine the virus titer by western blotting and plaque formation assays, respectively, at 24 h post-infection (h.p.i.). To demonstrate the effect of EGCG on PEDV attachment, the cells were washed with cold PBS three times and then collected for measurement of the PEDV ORF3 mRNA expression levels by qRT-PCR.

#### Entry Assay

Vero cells were infected with PEDV HLJBY at 4°C for 1 h, followed by washing with cold PBS three times. The cells were maintained in 2% DMEM containing different concentrations of EGCG (0, 20, 50, 100 μm) at 37°C for 1 h. After washing three times with the citric acid solution (40 mm citric acid, 10 mm KCl, 135 mm NaCl, pH 3.0) to remove un-internalized virus particles (Hancock, et al., 2010; Wang, et al., 2014; Huan, et al., 2017). Vero cells were washed with PBS three times. Intracellular viral proteins and cell supernatants were collected at 24 h.p.i to detect any changes that occurred in PEDV N protein levels or virus titers using western blots and plaque formation assays, respectively. The same steps were used with the Vero cells when measuring the levels of PEDV ORF3 mRNA by qRT-PCR.

#### Replication, Assembly and Release of PEDV

Vero cells were infected with PEDV HLJBY at 37°C for 1 h, followed by washing and incubation in 2% DMEM containing EGCG. The cells were collected to check the level of PEDV N protein on western blots at 4 and 6 h.p.i.

Vero cells were incubated with PEDV HLJBY at 37°C for 1 h. The cells were washed three times in PBS and then incubated with 2% DMEM containing various concentrations of EGCG at 37°C. At 24 h.p.i., cells or cell supernatants were collected to determine the viral titers and the number of viral RNA copies using a plaque formation assay and qRT-PCR, respectively.

### Western Blotting

The western blotting procedure followed previous descriptions (Huan, et al., 2017; Gao, et al., 2020). PEDV N and actin were detected using the above-mentioned primary antibody and a secondary antibody.

### Immunofluorescence Assays

IFAs were performed as per a previous description (Gao, et al., 2020). Briefly, Vero cells were fixed with 4% paraformaldehyde, permeabilized with 0.1% Triton X-100, blocked with 3% bovine serum albumin, and incubated with the anti-PEDV N antibody and an Alexa Fluor 5558-conjugated goat anti-rabbit secondary antibody (Invitrogen, United States). All images were taken at ×200 magnification.

### qRT-PCR

qRT-PCR was performed as described previously (Gao, et al., 2020). Total RNA was extracted, and gene expression was assessed by qRT-PCR using specific primers (PEDV-ORF3-F: TTT​GCA​CTG​TTT​AAA​GCG​TCT, PEDV-ORF3-R: AGT​AAA​AGC​AGA​CTA​AAC​AAA​GCC​T).

### Plaque Formation Assays

Viral culture supernatants were diluted from 10^−1^ to 10^−6^ in DMEM and then used to infect Vero cells seeded in 6-well plates at 37°C for 2 h before the DMEM was added to each well. Vero cells were cultured at 37°C with 5% CO_2_ for 3 days, after which they were stained with 0.5% crystal violet.

### Calculation of EC50 and IC50

EGCG was serially diluted to 100, 80, 50, 20, 10 and 5 μm, and was added to Vero cells which were infected with PEDV (MOI = 0.1). EGCG was present during the different stages of PEDV infection. The infected cells without EGCG treatment were set as mock control. We determined ORF3 mRNA levels at 24 h.p.i. by qRT-PCR and the values of inhibition were calculated as (1-ORF3 mRNA (compound)/ORF3 mRNA (mock)) × 100%. The values of inhibition and EGCG concentration were used to establish a dose response curve and further calculated the EC50 (concentration for 50% of maximal effect). The values of vitality were calculated as ORF3 mRNA (compound)/ORF3 mRNA (mock) × 100%. The values of vitality and EGCG concentration were used to establish a dose response curve and further calculate the IC50.

### Statistical Analysis

All experiments were independently repeated at least three times and all data were presented as means ± SD. All the data were analyzed by one-way ANOVA using the SPSS 17.0 software package. When the *p* values were less than 0.05, the differences were considered to be statistically significant (*p < 0.05 and **p < 0.01).

## Discussion

EGCG is known to exert its anti-infective effect on bacteria, viruses, and different fungal species (Steinmann, et al., 2013). It does this with hepatitis C virus (HCV) by inhibiting its entry by interfering with its binding to the target cells (Ciesek, et al., 2011; Calland, et al., 2012; Chen, et al., 2012). EGCG reduces hepatitis B virus (HBV) infection by decreasing HBV antigen expression, and decreasing the levels of extracellular HBV DNA and covalently closed circular DNA (Xu, et al., 2008; He, et al., 2011). EGCG possibly damages and inactivates the virions of herpes simplex virus 1 and 2 by binding to their envelope proteins (Isaacs, et al., 2008; Isaacs, et al., 2011). EGCG was found to inhibit transcription of the immediate-early genes (Rta, Zta and EA-D) of Epstein–Barr virus (Chang, et al., 2003). But the effect of EGCG on PEDV has awaited elucidation. Therefore, we explored the activity of EGCG against PEDV.

SARS coronavirus attaching and entering Vero E6 cells needs 30 –60 min, and extracellular virus particles were checked at 5 – 6 h.p.i. (Ng, et al., 2003a; Ng, et al., 2003b; Qinfen, et al., 2004; Hartenian, et al., 2020). Furthermore, one replication cycle of Porcine deltacoronavirus (PDCoV) takes 5–6 h (Qin, et al., 2019). In addition, we checked the life cycle of PEDV (MOI = 1) in Vero cells. Infectious progeny virus was detected in the supernatants at 6 h at a low titer (1.6 × 10^2^ PFU/ml), and progeny virus increased. The result revealed that the first generation of progeny virus were released at about 5 h.p.i., and one life cycle of PEDV takes about 6 h to complete. In this study, we showed that EGCG inhibited two different PEDV strains in Vero cells (Figures 1, 2), and went on to elucidate the anti-PEDV mechanism of EGCG. To investigate the effect of EGCG against PEDV infection, we first explored its effect on PEDV HLJBY binding. The results showed that EGCG reduced the expression of the N protein of PEDV and also reduced PEDV ORF3 mRNA levels and PEDV titers (Figure 3) in the binding assay, revealing that EGCG significantly reduced PEDV HLJBY attachment to Vero cells. These results are consistent with the findings from another study where EGCG inhibited the binding of human immunodeficiency virus 1 (Williamson, et al., 2006; Nance, et al., 2009; Jiang, et al., 2010; Li, et al., 2011). EGCG potently inhibited PEDV infection by preventing PEDV attachment to Vero cells. Therefore, EGCG may provide a new way of preventing PEDV infections. We next explored the effect of EGCG on PEDV HLJBY entry into Vero cells. We observed that EGCG decreased the levels of the PEDV N protein, PEDV ORF3 mRNA and the PEDV titer using an entry assay (Figure 4). Other studies have shown that EGCG can block influenza virus entry of cells by binding to hemagglutinin (Nakayama, et al., 1993; Imanishi, et al., 2002; Song, et al., 2005). Our results indicate that EGCG was able to inhibit PEDV infection by blocking PEDV entry into Vero cells.

EGCG has been reported to suppress enterovirus replication (Ho, et al., 2009). Therefore, we investigated the effect of EGCG on PEDV HLJBY replication. We found that EGCG reduced PEDV replication, but this effect was inferior to that seen with PEDV attachment and entry. This result shows that EGCG has potential to inhibit PEDV replication. Although we discovered that EGCG treatment was able to decrease PEDV assembly, it had no effect on PEDV release.

In conclusion, we have provided evidence that EGCG inhibited PEDV infection by blocking viral attachment, entry, replication and assembly. Although The bioavailability of EGCG is usually poor (about 4.5–7.2%) (Del Rio, et al., 2010), EGCG contribute to the health benefits (Chen, et al., 1997; Sabu, et al., 2002; Henning, et al., 2004; Yang and Wang, 2016). Chow HH. revealed repeated intake of catechins in humans increased bioavailability of EGCG (Chow, et al., 2003). In addition, the increase in EGCG bioavailability in mice fed the catechin diet for two weeks was reported (Ishii, et al., 2019). In previous studies, EGCG had similar antiviral activity against various viruses, EGCG against PRRSV with an EC50 of 48.2–63.09 µm *in vitro* (Ge, et al., 2018), and EGCG inhibited JEV with an IC50 of 4.9–20 µm *in vitro* (Wang, et al., 2018). EGCG against PCV2 *in vitro* with EC50 was 37.79 ± 1.64 µm (Li, et al., 2020). In our study, the EC50 or IC50 of EGCG against PEDV *in vitro* was12.39–83.18 µm.

## Data Availability

The original contributions presented in the study are included in the article/Supplementary Material, further inquiries can be directed to the corresponding author.
